# *N*-(4-Methoxy-2-nitrophenyl)-2-(3-methyl-2-oxo-1,2-dihydroquinoxalin-1-yl)acetamide

**DOI:** 10.1107/S2414314623001918

**Published:** 2023-03-15

**Authors:** Mohcine Missioui, Joel T. Mague, Abdulsalam Alsubari, Mhammed Ansar, El Mokhtar Essassi, Youssef Ramli

**Affiliations:** a Laboratory of Medicinal Chemistry, Drug Sciences Research Center, Faculty of Medicine and Pharmacy, Mohammed V University in Rabat, Morocco; b Department of Chemistry, Tulane University, New Orleans, LA 70118, USA; c Laboratory of Medicinal Chemistry, Faculty of Clinical Pharmacy, 21 September University, Yemen; d Laboratory of Heterocyclic Organic Chemistry, Faculty of Sciences, Mohammed V, University, Rabat, Morocco; University of Aberdeen, United Kingdom

**Keywords:** crystal structure, hydrogen bond, acetamide, quinoxaline, π-stacking, C=O⋯π inter­action

## Abstract

The quinoxaline unit in the title mol­ecule, C_18_H_16_N_4_O_5_, is slightly puckered [dihedral angle between the rings = 2.07 (12)°] while the whole mol­ecule adopts an L-shaped conformation. Intra­molecular hydrogen bonding determines the orientation of the substituted phenyl ring and the amide nitro­gen atom is almost planar. The packing in the crystal is governed by C—H⋯O hydrogen bonds and slipped π-stacking inter­actions.

## Abstract 2

The quinoxaline unit in the title mol­ecule is slightly puckered [dihedral angle between the rings = 2.07 (12)°] while the whole mol­ecule adopts an L-shaped conformation. The packing in the crystal is governed by C—H⋯O hydrogen bonds and slipped π-stacking inter­actions.

## Graphical abstract

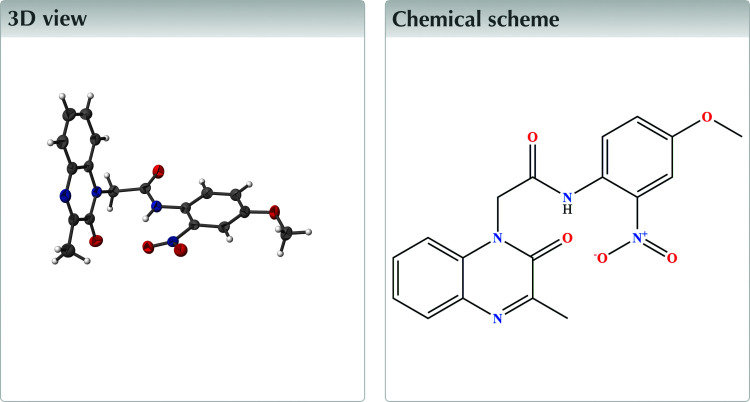

## Structure description

Quinoxaline and its derivatives have attracted significant considerations because of their pharmacological activities (*e.g.*, Abad *et al.*, 2021 ▸) and industrial properties (*e.g.*, Laabaissi *et al.*, 2020 ▸). As a continuation of our work on the synthesis of 3-methyl­quinoxalin-2-one derivatives in order to evaluate their pharmacological activities (Ramli *et al.* 2018 ▸), the title compound, C_18_H_16_N_4_O_5_ was synthesized and its crystal structure is reported here.

**Figure 1:**
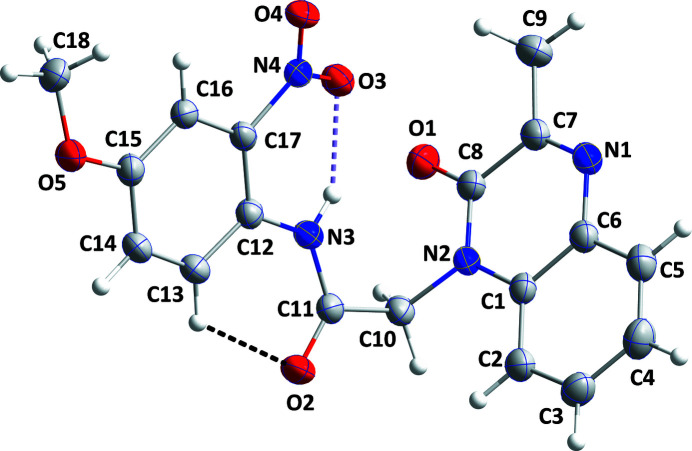
The title mol­ecule showing 50% displacement ellipsoids. The intra­molecular hydrogen bonds are depicted by dashed lines.

The mol­ecule adopts an L-shaped conformation with atom C10 at the apex of the ‘L’ (Fig. 1 ▸). The orientation of the pendant 2-nitro-4-meth­oxy­phenyl ring is primarily determined by the strong intra­molecular N3—H3*A*⋯O1 and weaker C13—H13⋯O2 hydrogen bonds (Table 1 ▸). H3*A* may also participate in hydrogen bonding with O1, but this is a weak inter­action at best due to the long H3*A*⋯O1 separation (Table 1 ▸). The quinoxaline unit is not quite planar, as indicated by the dihedral angle of 2.07 (12)° between the mean planes of the constituent rings. The dihedral angle between the mean planes of the C12–C17 and the C1/C6/N1/C7/C8/N2 rings is 81.96 (5)°. The sum of the inter­bond angles about N3 is 360°, which may be inter­preted as the participation of its lone pair in π bonding: this is supported by the N3—C11 and N3—C12 bond distances of 1.358 (2) and 1.408 (2) Å, respectively, which are shorter than would be expected for *sp* ^2^(C)—sp^3^(N) bonds.

**Figure 2:**
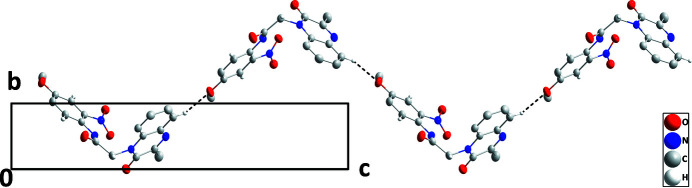
A portion of an [001] chain viewed along the *a*-axis direction with C—H⋯O hydrogen bonds depicted by dashed lines Non-inter­acting hydrogen atoms are omitted for clarity.

**Figure 3:**
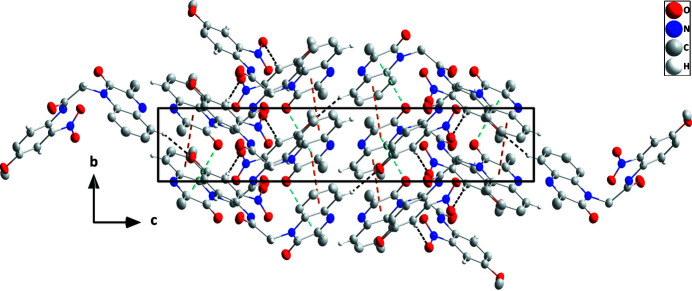
Packing viewed along the *a*-axis direction with C—H⋯O hydrogen bonds depicted by black dashed lines. Slipped π-stacking and C=O⋯π(ring) inter­actions are depicted, respectively, by orange and light-blue dashed lines. Non-inter­acting hydrogen atoms are omitted for clarity.

In the crystal, C5—H5⋯O5 hydrogen bonds (Table 1 ▸) form zigzag chains of mol­ecules extending along the *c*-axis direction (Fig. 2 ▸). The chains are stacked along the *b*-axis direction through slipped π-stacking inter­actions between the C1–C6 and C1/C6/N1/C7/C8/N2 rings [centroid–centroid separation = 3.6684 (12) Å, dihedral angle = 2.07 (10)°, slippage alternates between 1.40 and 1.28 Å along the stack]. The π-stacking is reinforced by C8=O1⋯*Cg*1 inter­actions, where *Cg*1 is the centroid of the C1/C6/N1/C7/C8/N2 ring: O1⋯*Cg*1 = 3.3333 (16) Å, C8⋯*Cg*1 = 3.689 (2) Å, C8=O1⋯*Cg*1 = 96.89 (2)°. The stacks are linked by C10—H10*A*⋯O2 and C16—H16⋯O4 hydrogen bonds (Table 1 ▸), generating a three-dimensional network (Fig. 3 ▸).

## Synthesis and crystallization

A mass of 1.00 g (6.24 mmol) of 3-methyl­quinoxalin-2(1*H*)-one was dissolved in 25 ml of di­methyl­formamide, then 1.53 g (6.24 mmol) of 2-chloro-*N*-(4-meth­oxy-2-nitro­phen­yl)acetamide were added followed by 1.0 g (7.5 mmol) of potassium bicarbonate, and a spatula tip of BTBA [2-benzyl­sulfanyl-5-(tri­fluoro­meth­yl)benzoic acid] was used for the phase-transfer catalysis. The reaction was stirred for 2 h under reflux at 80°C. When the starting reagents had completely reacted, 500 ml of distilled water were added and a few minutes later the product precipitated. This was filtered, dried and recrystallized from hot ethanol solution to yield light-yellow plate-like crystals of the title compound.

## Refinement

Crystal data, data collection and structure refinement details are summarized in Table 2 ▸.

## Supplementary Material

Crystal structure: contains datablock(s) global, I. DOI: 10.1107/S2414314623001918/hb4426sup1.cif

Structure factors: contains datablock(s) I. DOI: 10.1107/S2414314623001918/hb4426Isup2.hkl

Supporting information file. DOI: 10.1107/S2414314623001918/hb4426Isup3.cml

CCDC reference: 2245645

Additional supporting information:  crystallographic information; 3D view; checkCIF report

## Figures and Tables

**Table 1:** Hydrogen-bond geometry (Å, °)

*D*—H⋯*A*	*D*—H	H⋯*A*	*D*⋯*A*	*D*—H⋯*A*
N3—H3*A*⋯O1	0.85 (2)	2.55 (2)	3.245 (2)	139 (2)
N3—H3*A*⋯O3	0.85 (2)	1.97 (2)	2.655 (2)	137 (2)
C5—H5⋯O5^i^	0.95	2.43	3.331 (2)	159
C10—H10*A*⋯O2^ii^	0.99	2.37	3.306 (2)	157
C13—H13⋯O2	0.95	2.21	2.842 (2)	123
C16—H16⋯O4^iii^	0.95	2.49	3.401 (2)	162

Symmetry codes: (i) 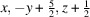 ; (ii) 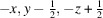 ; (iii) 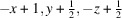 .

**Table 2:** Experimental details

Crystal data	
Chemical formula	C_18_H_16_N_4_O_5_
*M*_r_	368.35
Crystal system, space group	Monoclinic, *P*2_1_/*c*
Temperature (K)	150
*a*, *b*, *c* (Å)	15.8241 (3), 4.4930 (1), 23.6480 (5)
β (°)	103.018 (1)
*V* (Å^3^)	1638.11 (6)
*Z*	4
Radiation type	Cu *K*α
μ (mm^−1^)	0.94
Crystal size (mm)	0.12 × 0.08 × 0.02

Data collection	
Diffractometer	Bruker D8 VENTURE PHOTON 3 CPAD
Absorption correction	Multi-scan (*SADABS*; Krause *et al.*, 2015 ▸)
*T*_min_, *T*_max_	0.89, 0.98
No. of measured, independent and observed [*I* > 2σ(*I*)] reflections	22301, 3119, 2270
*R*_int_	0.074
(sin θ/λ)_max_ (Å^−1^)	0.610

Refinement	
*R*[*F* ^2^ > 2σ(*F* ^2^)], *wR*(*F* ^2^), *S*	0.041, 0.109, 1.02
No. of reflections	3119
No. of parameters	250
H-atom treatment	H atoms treated by a mixture of independent and constrained refinement
Δρ_max_, Δρ_min_ (e Å^−3^)	0.20, −0.21

Computer programs: *APEX4* and *SAINT* (Bruker, 2021 ▸), *SHELXT* (Sheldrick, 2015*a* ▸), *SHELXL2018/1* (Sheldrick, 2015*b* ▸), *DIAMOND* (Brandenburg & Putz, 2012 ▸) and *SHELXTL* (Sheldrick, 2008 ▸).

## full crystallographic data

### Crystal data

**Table:**

C_18_H_16_N_4_O_5_	*F*(000) = 768
*M**_r_* = 368.35	*D*_x_ = 1.494 Mg m^−^^3^
Monoclinic, *P*2_1_/*c*	Cu *K*α radiation, λ = 1.54178 Å
*a* = 15.8241 (3) Å	Cell parameters from 7216 reflections
*b* = 4.4930 (1) Å	θ = 2.9–70.2°
*c* = 23.6480 (5) Å	µ = 0.94 mm^−^^1^
β = 103.018 (1)°	*T* = 150 K
*V* = 1638.11 (6) Å^3^	Plate, light yellow
*Z* = 4	0.12 × 0.08 × 0.02 mm

### Data collection

**Table:**

Bruker D8 VENTURE PHOTON 3 CPAD diffractometer	3119 independent reflections
Radiation source: INCOATEC IµS micro—-focus source	2270 reflections with *I* > 2σ(*I*)
Mirror monochromator	*R*_int_ = 0.074
Detector resolution: 7.3910 pixels mm^-1^	θ_max_ = 70.1°, θ_min_ = 2.9°
φ and ω scans	*h* = −19→19
Absorption correction: multi-scan (*SADABS*; Krause *et al.*, 2015)	*k* = −5→5
*T*_min_ = 0.89, *T*_max_ = 0.98	*l* = −28→28
22301 measured reflections	

### Refinement

**Table:**

Refinement on *F*^2^	Primary atom site location: dual
Least-squares matrix: full	Secondary atom site location: difference Fourier map
*R*[*F*^2^ > 2σ(*F*^2^)] = 0.041	Hydrogen site location: mixed
*wR*(*F*^2^) = 0.109	H atoms treated by a mixture of independent and constrained refinement
*S* = 1.01	*w* = 1/[σ^2^(*F*_o_^2^) + (0.046*P*)^2^ + 0.6927*P*] where *P* = (*F*_o_^2^ + 2*F*_c_^2^)/3
3119 reflections	(Δ/σ)_max_ < 0.001
250 parameters	Δρ_max_ = 0.20 e Å^−^^3^
0 restraints	Δρ_min_ = −0.21 e Å^−^^3^

### Special details

**Table:**

Experimental. The diffraction data were obtained from 10 sets of frames, each of width 0.5° in ω or φ, collected with scan parameters determined by the "strategy" routine in *APEX4*. The scan time was θ-dependent and ranged from 10 to 30 sec/frame.
Geometry. All esds (except the esd in the dihedral angle between two l.s. planes) are estimated using the full covariance matrix. The cell esds are taken into account individually in the estimation of esds in distances, angles and torsion angles; correlations between esds in cell parameters are only used when they are defined by crystal symmetry. An approximate (isotropic) treatment of cell esds is used for estimating esds involving l.s. planes.
Refinement. Refinement of F^2^ against ALL reflections. The weighted R-factor wR and goodness of fit S are based on F^2^, conventional R-factors R are based on F, with F set to zero for negative F^2^. The threshold expression of F^2^ > 2sigma(F^2^) is used only for calculating R-factors(gt) etc. and is not relevant to the choice of reflections for refinement. R-factors based on F^2^ are statistically about twice as large as those based on F, and R- factors based on ALL data will be even larger. H-atoms attached to carbon were placed in calculated positions (C—H = 0.95 - 0.99 Å) and were included as riding contributions with isotropic displacement parameters 1.2 - 1.5 times those of the attached atoms. That attached to nitrogen was placed in a location derived from a difference map and refined independently.

### Fractional atomic coordinates and isotropic or equivalent isotropic displacement parameters (Å^2^)

**Table:**

	*x*	*y*	*z*	*U*_iso_*/*U*_eq_	
O1	0.29763 (9)	−0.0001 (3)	0.34072 (6)	0.0396 (4)	
O2	0.06996 (9)	0.4993 (4)	0.21704 (6)	0.0420 (4)	
O3	0.38691 (9)	0.5131 (3)	0.29319 (6)	0.0344 (3)	
O4	0.45916 (8)	0.9029 (3)	0.27884 (6)	0.0341 (3)	
O5	0.30452 (9)	1.3237 (3)	0.09025 (5)	0.0374 (4)	
N1	0.30123 (10)	0.4946 (4)	0.45836 (6)	0.0334 (4)	
N2	0.18929 (10)	0.3026 (4)	0.35555 (6)	0.0270 (3)	
N3	0.21772 (10)	0.5104 (4)	0.24679 (7)	0.0292 (4)	
H3A	0.2615 (15)	0.437 (5)	0.2704 (10)	0.042 (7)*	
N4	0.39485 (10)	0.7433 (4)	0.26623 (6)	0.0278 (4)	
C1	0.15864 (12)	0.5072 (4)	0.39079 (7)	0.0278 (4)	
C2	0.07504 (13)	0.6245 (5)	0.37620 (8)	0.0323 (4)	
H2	0.036760	0.570160	0.340692	0.039*	
C3	0.04812 (14)	0.8202 (5)	0.41361 (9)	0.0381 (5)	
H3	−0.009040	0.898860	0.403726	0.046*	
C4	0.10365 (15)	0.9037 (5)	0.46563 (9)	0.0420 (5)	
H4	0.084266	1.036862	0.491251	0.050*	
C5	0.18659 (14)	0.7926 (5)	0.47968 (8)	0.0367 (5)	
H5	0.224600	0.851568	0.514975	0.044*	
C6	0.21584 (12)	0.5946 (5)	0.44297 (8)	0.0304 (4)	
C7	0.32731 (12)	0.3047 (5)	0.42509 (8)	0.0312 (4)	
C8	0.27135 (12)	0.1856 (5)	0.37100 (8)	0.0300 (4)	
C9	0.41867 (13)	0.1955 (6)	0.43809 (9)	0.0436 (5)	
H9A	0.419326	−0.020208	0.444301	0.065*	
H9B	0.451685	0.293823	0.473152	0.065*	
H9C	0.445009	0.241173	0.405358	0.065*	
C10	0.13459 (12)	0.2067 (5)	0.29953 (7)	0.0298 (4)	
H10A	0.073965	0.186274	0.303628	0.036*	
H10B	0.154343	0.008574	0.289498	0.036*	
C11	0.13691 (12)	0.4224 (4)	0.25020 (8)	0.0287 (4)	
C12	0.24079 (12)	0.7179 (4)	0.20827 (7)	0.0269 (4)	
C13	0.18016 (12)	0.8268 (5)	0.16010 (8)	0.0305 (4)	
H13	0.121703	0.760845	0.153328	0.037*	
C14	0.20368 (12)	1.0275 (5)	0.12241 (8)	0.0318 (4)	
H14	0.161097	1.098298	0.090255	0.038*	
C15	0.28865 (12)	1.1285 (5)	0.13054 (8)	0.0299 (4)	
C16	0.34992 (12)	1.0312 (4)	0.17832 (7)	0.0279 (4)	
H16	0.407976	1.101331	0.185046	0.033*	
C17	0.32553 (12)	0.8291 (4)	0.21649 (7)	0.0263 (4)	
C18	0.39238 (13)	1.4007 (5)	0.09209 (9)	0.0391 (5)	
H18A	0.425519	1.219924	0.088668	0.059*	
H18B	0.417397	1.499676	0.128995	0.059*	
H18C	0.394687	1.535425	0.059889	0.059*	

### Atomic displacement parameters (Å^2^)

**Table:**

	*U*^11^	*U*^22^	*U*^33^	*U*^12^	*U*^13^	*U*^23^
O1	0.0387 (8)	0.0464 (9)	0.0348 (7)	0.0053 (7)	0.0106 (6)	−0.0050 (7)
O2	0.0261 (7)	0.0629 (11)	0.0347 (7)	−0.0013 (7)	0.0018 (6)	0.0116 (7)
O3	0.0320 (7)	0.0373 (8)	0.0321 (7)	0.0016 (6)	0.0035 (6)	0.0065 (6)
O4	0.0247 (7)	0.0410 (8)	0.0341 (7)	−0.0036 (6)	0.0015 (5)	−0.0004 (6)
O5	0.0317 (7)	0.0511 (9)	0.0287 (7)	−0.0024 (7)	0.0054 (6)	0.0115 (6)
N1	0.0328 (9)	0.0399 (10)	0.0261 (8)	−0.0030 (8)	0.0036 (7)	0.0027 (7)
N2	0.0266 (8)	0.0320 (9)	0.0216 (7)	−0.0024 (7)	0.0041 (6)	−0.0005 (6)
N3	0.0256 (8)	0.0338 (9)	0.0264 (8)	−0.0010 (7)	0.0025 (7)	0.0025 (7)
N4	0.0255 (8)	0.0332 (9)	0.0248 (7)	0.0027 (7)	0.0058 (6)	−0.0008 (7)
C1	0.0316 (10)	0.0296 (10)	0.0233 (8)	−0.0026 (8)	0.0087 (7)	0.0008 (8)
C2	0.0328 (10)	0.0359 (11)	0.0283 (9)	0.0004 (9)	0.0070 (8)	0.0038 (8)
C3	0.0369 (11)	0.0408 (12)	0.0388 (11)	0.0078 (9)	0.0131 (9)	0.0040 (9)
C4	0.0505 (13)	0.0428 (13)	0.0362 (11)	0.0038 (11)	0.0170 (10)	−0.0041 (10)
C5	0.0440 (12)	0.0387 (12)	0.0273 (10)	−0.0031 (10)	0.0082 (9)	−0.0019 (9)
C6	0.0328 (10)	0.0332 (11)	0.0252 (9)	−0.0028 (8)	0.0068 (8)	0.0015 (8)
C7	0.0300 (10)	0.0371 (11)	0.0265 (9)	−0.0014 (9)	0.0061 (8)	0.0041 (8)
C8	0.0292 (10)	0.0351 (11)	0.0262 (9)	0.0008 (8)	0.0076 (8)	0.0029 (8)
C9	0.0316 (11)	0.0577 (15)	0.0388 (11)	0.0026 (10)	0.0024 (9)	0.0035 (10)
C10	0.0290 (10)	0.0347 (11)	0.0250 (9)	−0.0052 (8)	0.0045 (7)	−0.0028 (8)
C11	0.0276 (10)	0.0339 (11)	0.0240 (9)	−0.0010 (8)	0.0048 (8)	−0.0043 (8)
C12	0.0290 (9)	0.0288 (10)	0.0228 (8)	−0.0021 (8)	0.0057 (7)	−0.0024 (7)
C13	0.0256 (9)	0.0384 (12)	0.0256 (9)	−0.0013 (8)	0.0019 (8)	−0.0016 (8)
C14	0.0284 (10)	0.0407 (12)	0.0237 (9)	0.0012 (9)	0.0005 (7)	0.0010 (8)
C15	0.0331 (10)	0.0333 (11)	0.0233 (9)	−0.0002 (8)	0.0067 (8)	0.0007 (8)
C16	0.0259 (9)	0.0334 (11)	0.0243 (9)	−0.0009 (8)	0.0054 (7)	−0.0032 (8)
C17	0.0268 (9)	0.0302 (10)	0.0212 (8)	0.0042 (8)	0.0039 (7)	−0.0019 (7)
C18	0.0346 (11)	0.0513 (14)	0.0332 (10)	−0.0022 (10)	0.0113 (9)	0.0064 (10)

### Geometric parameters (Å, º)

**Table:**

O1—C8	1.231 (2)	C5—C6	1.393 (3)
O2—C11	1.218 (2)	C5—H5	0.9500
O3—N4	1.236 (2)	C7—C8	1.482 (3)
O4—N4	1.226 (2)	C7—C9	1.492 (3)
O5—C15	1.360 (2)	C9—H9A	0.9800
O5—C18	1.424 (2)	C9—H9B	0.9800
N1—C7	1.290 (3)	C9—H9C	0.9800
N1—C6	1.392 (3)	C10—C11	1.523 (3)
N2—C8	1.372 (2)	C10—H10A	0.9900
N2—C1	1.399 (2)	C10—H10B	0.9900
N2—C10	1.474 (2)	C12—C13	1.402 (3)
N3—C11	1.358 (2)	C12—C17	1.403 (3)
N3—C12	1.408 (2)	C13—C14	1.377 (3)
N3—H3A	0.85 (2)	C13—H13	0.9500
N4—C17	1.468 (2)	C14—C15	1.391 (3)
C1—C2	1.393 (3)	C14—H14	0.9500
C1—C6	1.412 (3)	C15—C16	1.383 (3)
C2—C3	1.381 (3)	C16—C17	1.395 (3)
C2—H2	0.9500	C16—H16	0.9500
C3—C4	1.393 (3)	C18—H18A	0.9800
C3—H3	0.9500	C18—H18B	0.9800
C4—C5	1.373 (3)	C18—H18C	0.9800
C4—H4	0.9500		
			
C15—O5—C18	117.94 (15)	H9A—C9—H9B	109.5
C7—N1—C6	118.63 (16)	C7—C9—H9C	109.5
C8—N2—C1	121.83 (15)	H9A—C9—H9C	109.5
C8—N2—C10	117.15 (15)	H9B—C9—H9C	109.5
C1—N2—C10	121.01 (15)	N2—C10—C11	113.08 (15)
C11—N3—C12	128.05 (16)	N2—C10—H10A	109.0
C11—N3—H3A	119.0 (16)	C11—C10—H10A	109.0
C12—N3—H3A	113.0 (16)	N2—C10—H10B	109.0
O4—N4—O3	122.64 (15)	C11—C10—H10B	109.0
O4—N4—C17	118.10 (16)	H10A—C10—H10B	107.8
O3—N4—C17	119.25 (15)	O2—C11—N3	124.97 (18)
C2—C1—N2	122.56 (17)	O2—C11—C10	120.42 (17)
C2—C1—C6	119.82 (18)	N3—C11—C10	114.60 (16)
N2—C1—C6	117.62 (17)	C13—C12—C17	116.49 (17)
C3—C2—C1	119.66 (18)	C13—C12—N3	121.81 (17)
C3—C2—H2	120.2	C17—C12—N3	121.70 (16)
C1—C2—H2	120.2	C14—C13—C12	121.29 (17)
C2—C3—C4	120.9 (2)	C14—C13—H13	119.4
C2—C3—H3	119.6	C12—C13—H13	119.4
C4—C3—H3	119.6	C13—C14—C15	121.20 (17)
C5—C4—C3	119.6 (2)	C13—C14—H14	119.4
C5—C4—H4	120.2	C15—C14—H14	119.4
C3—C4—H4	120.2	O5—C15—C16	124.75 (17)
C4—C5—C6	121.01 (19)	O5—C15—C14	116.04 (16)
C4—C5—H5	119.5	C16—C15—C14	119.20 (18)
C6—C5—H5	119.5	C15—C16—C17	119.27 (17)
N1—C6—C5	118.84 (17)	C15—C16—H16	120.4
N1—C6—C1	122.14 (17)	C17—C16—H16	120.4
C5—C6—C1	119.01 (18)	C16—C17—C12	122.51 (16)
N1—C7—C8	123.66 (17)	C16—C17—N4	115.08 (16)
N1—C7—C9	121.36 (18)	C12—C17—N4	122.41 (16)
C8—C7—C9	114.95 (18)	O5—C18—H18A	109.5
O1—C8—N2	121.88 (17)	O5—C18—H18B	109.5
O1—C8—C7	122.16 (17)	H18A—C18—H18B	109.5
N2—C8—C7	115.91 (17)	O5—C18—H18C	109.5
C7—C9—H9A	109.5	H18A—C18—H18C	109.5
C7—C9—H9B	109.5	H18B—C18—H18C	109.5
			
C8—N2—C1—C2	−178.95 (18)	C8—N2—C10—C11	−95.9 (2)
C10—N2—C1—C2	1.9 (3)	C1—N2—C10—C11	83.3 (2)
C8—N2—C1—C6	1.0 (3)	C12—N3—C11—O2	4.7 (3)
C10—N2—C1—C6	−178.14 (16)	C12—N3—C11—C10	−176.44 (17)
N2—C1—C2—C3	178.42 (18)	N2—C10—C11—O2	−133.59 (19)
C6—C1—C2—C3	−1.5 (3)	N2—C10—C11—N3	47.5 (2)
C1—C2—C3—C4	0.5 (3)	C11—N3—C12—C13	−11.4 (3)
C2—C3—C4—C5	0.7 (3)	C11—N3—C12—C17	167.84 (18)
C3—C4—C5—C6	−0.7 (3)	C17—C12—C13—C14	1.4 (3)
C7—N1—C6—C5	178.03 (18)	N3—C12—C13—C14	−179.36 (18)
C7—N1—C6—C1	−3.4 (3)	C12—C13—C14—C15	0.4 (3)
C4—C5—C6—N1	178.24 (19)	C18—O5—C15—C16	9.8 (3)
C4—C5—C6—C1	−0.4 (3)	C18—O5—C15—C14	−171.35 (18)
C2—C1—C6—N1	−177.06 (18)	C13—C14—C15—O5	179.29 (18)
N2—C1—C6—N1	3.0 (3)	C13—C14—C15—C16	−1.8 (3)
C2—C1—C6—C5	1.5 (3)	O5—C15—C16—C17	−179.77 (18)
N2—C1—C6—C5	−178.47 (17)	C14—C15—C16—C17	1.4 (3)
C6—N1—C7—C8	0.0 (3)	C15—C16—C17—C12	0.4 (3)
C6—N1—C7—C9	178.12 (18)	C15—C16—C17—N4	−179.10 (16)
C1—N2—C8—O1	178.15 (18)	C13—C12—C17—C16	−1.7 (3)
C10—N2—C8—O1	−2.7 (3)	N3—C12—C17—C16	178.99 (17)
C1—N2—C8—C7	−4.1 (3)	C13—C12—C17—N4	177.70 (16)
C10—N2—C8—C7	175.10 (16)	N3—C12—C17—N4	−1.6 (3)
N1—C7—C8—O1	−178.52 (19)	O4—N4—C17—C16	18.3 (2)
C9—C7—C8—O1	3.2 (3)	O3—N4—C17—C16	−160.85 (16)
N1—C7—C8—N2	3.7 (3)	O4—N4—C17—C12	−161.20 (17)
C9—C7—C8—N2	−174.54 (17)	O3—N4—C17—C12	19.7 (3)

### Hydrogen-bond geometry (Å, º)

**Table:**

*D*—H···*A*	*D*—H	H···*A*	*D*···*A*	*D*—H···*A*
N3—H3*A*···O1	0.85 (2)	2.55 (2)	3.245 (2)	139 (2)
N3—H3*A*···O3	0.85 (2)	1.97 (2)	2.655 (2)	137 (2)
C5—H5···O5^i^	0.95	2.43	3.331 (2)	159
C10—H10*A*···O2^ii^	0.99	2.37	3.306 (2)	157
C13—H13···O2	0.95	2.21	2.842 (2)	123
C16—H16···O4^iii^	0.95	2.49	3.401 (2)	162

Symmetry codes: (i) *x*, −*y*+5/2, *z*+1/2; (ii) −*x*, *y*−1/2, −*z*+1/2; (iii) −*x*+1, *y*+1/2, −*z*+1/2.
